# Supplementary material for: The Molecular Subtype of Adult Acute Lymphoblastic Leukemia Samples Determines the Engraftment Site and Proliferation Kinetics in Patient-Derived Xenograft Models
Source: Cells. 2022 Jan 3;11(1):150. doi: 10.3390/cells11010150 (PMC8750004; doi:10.3390/cells11010150)
Supplement: Supplementary file 1 [file cells-11-00150-s001.zip › cells-1514928-supplementary.pdf]

Supplementary Figures

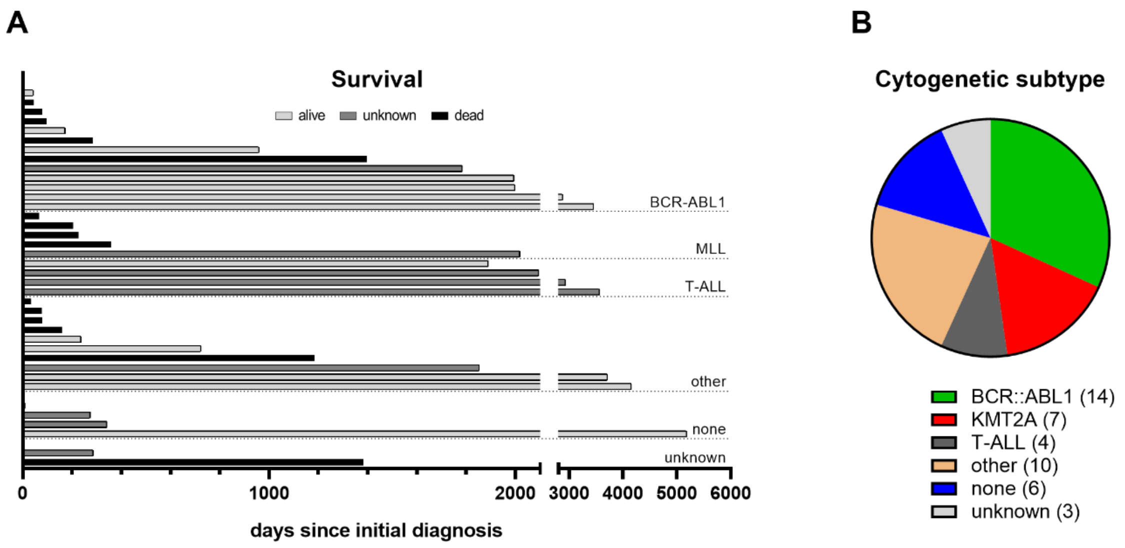

Figure S1. Survival and cytogenetic subgroups of the patient cohort. (**A**) The duration between the initial diagnosis and the last contact is displayed. Light grey, black and dark grey bars indicate that the patient is still alive, dead or that the last follow-up occurred more than one year ago (status unknown), respectively. Bar stacks are arranged by cytogenetic subtype as stated on the right hand side. Two patients were excluded from the graph due to lack of contact after sample collection. (**B**) Distribution of cytogenetic aberrations across the patient cohort. The total numbers of samples with the respective subtype is written in brackets.

pathogenic/likely pathogenic<br>uncertain<br>benign/likely benign<br>not described	#0122	#0094	#0134	#0159	#0031	#0054	#0074	#P67	#0152	#0181
FLT3 Asp835Val	3									
FLT3 Val592Ala		44	51							
KRAS Gly12Ser	31									
NRAS Gln61His				6						
PIK3CA Gln546Lys				4						
MET Thr1010Ile				51						
TP53 Arg283Ser		86								
JAK3 Val722Ile				51						
KDR Gln472His					100		50	48		
TP53 Pro72Arg	100	94	52	56		99	50	99	100	99
APC Ala1582Pro	4									
CDKN2A Ala132fs								4		
HRAS Val29fs									57	

Figure S2. Genetic characterization of eleven selected B-ALL patients using Ion AmpliSeq™ Cancer Hotspot Panel v2. All variants resulting in amino acid changes are displayed and ranked by pathogenicity based on their description in the ClinVar database. Mutations without database listing are depicted in grey as "not described". The numbers within the boxes describe the allele frequency with dark grey boxes indicating absence of the variant. Boxes are further color-coded to represent the allele frequency with low rates painted in red and high rates in blue.

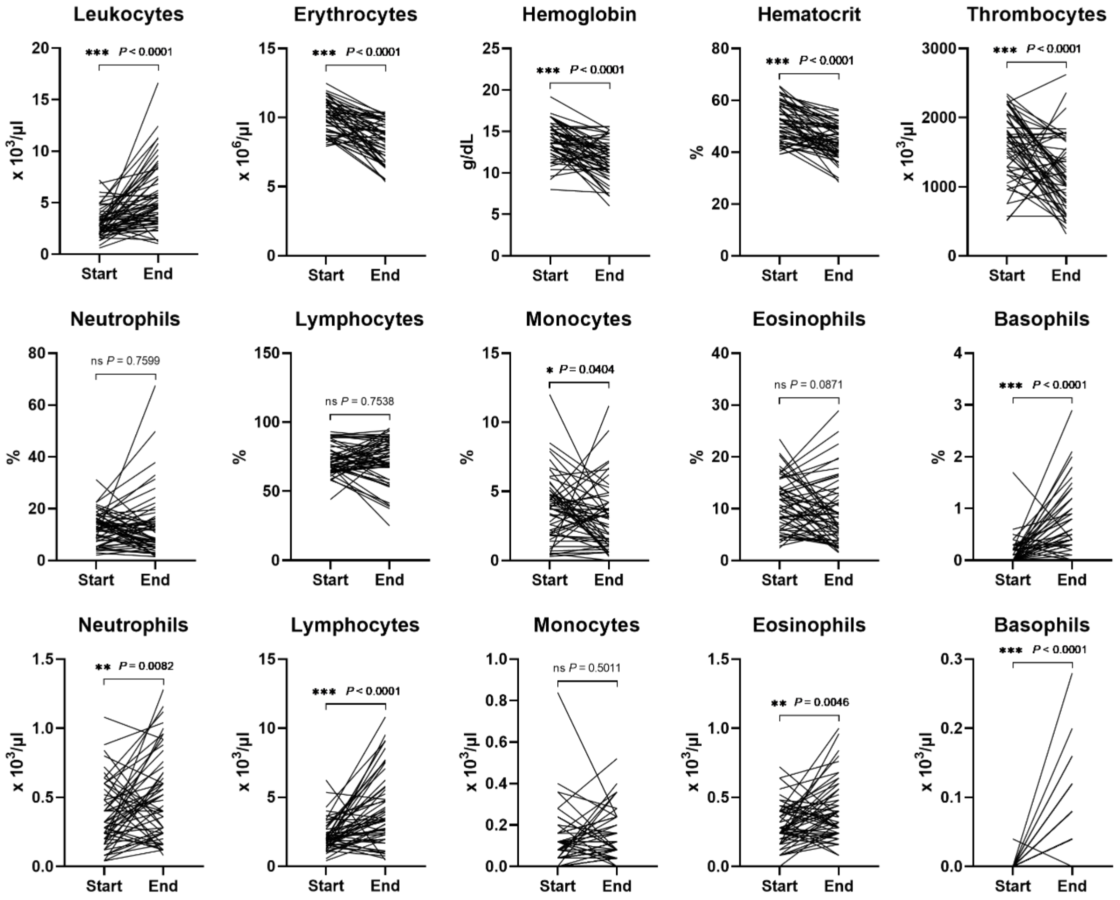

Figure S3. Influence of ALL cell xenotransplantation on murine blood counts. Blood count analysis of 58 representative mice injected with primary tumor material or tumor cells obtained from spleen or bone marrow of xenotransplanted animals. Basal "start" values were determined within two weeks of cell injection and "end" values were generally collected at the day of euthanasia or no earlier than three days before termination of the experiment. Wilcoxon test.

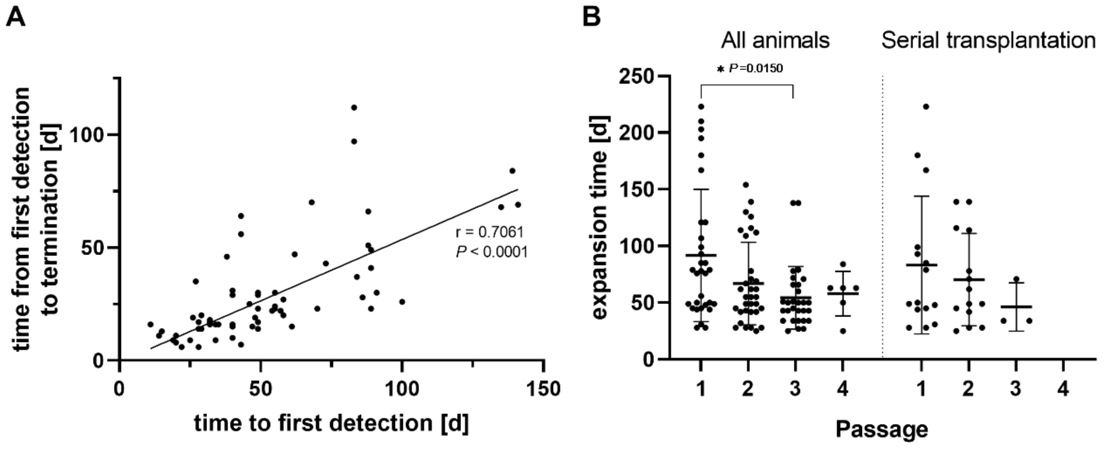

Figure S4. Correlation of time to first blast detection and influence of serial transplantation on growth kinetics. Each dot represents an individual animal. (A) Correlation of the time between tumor cell injection and first blast detection in peripheral blood, and the time between first detection and termination of the experiment. Flow cytometric determination of peripheral blood blast frequencies above 0.5% in combination with a visible distinct population were considered as positive engraftment. Spearman's correlation value r. (B) Evaluation of the influence of serial transplantation on the overall

expansion time in all animals used for tumor cell expansion (left panel) and only those animals whose cells were subsequently injected in the next xenograft passage (right panel). Mean ± standard deviation, Kruskal-Wallis and post-hoc Dunn's multiple comparisons test.

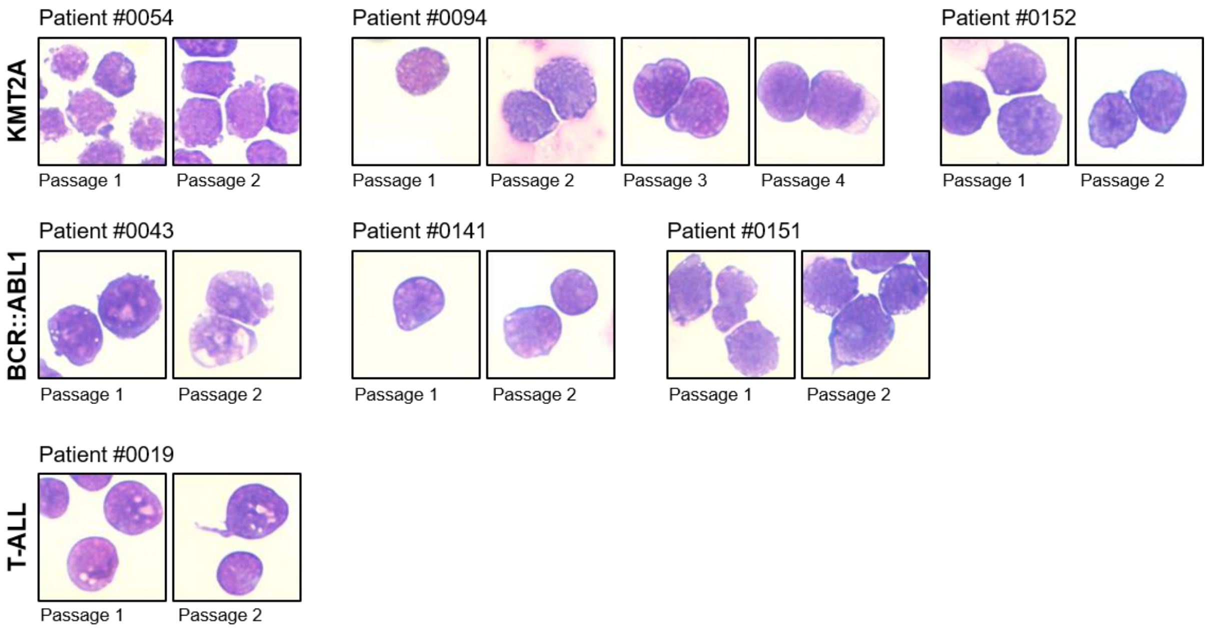

Figure S5. Blast morphology of selected *KMT2A* rearranged, *BCR::ABL1* translocated and T-ALL samples throughout serial transplantation. After expansion in xenograft mice, tumor cells were harvested from the bone marrow, spun onto microscopic slides and Pappenheim stained. Cytospin preparations of the first two passages (four passages for patient #0094) are shown. Representative cells were captured at 100-fold magnification using the EVOS xl core imaging system.

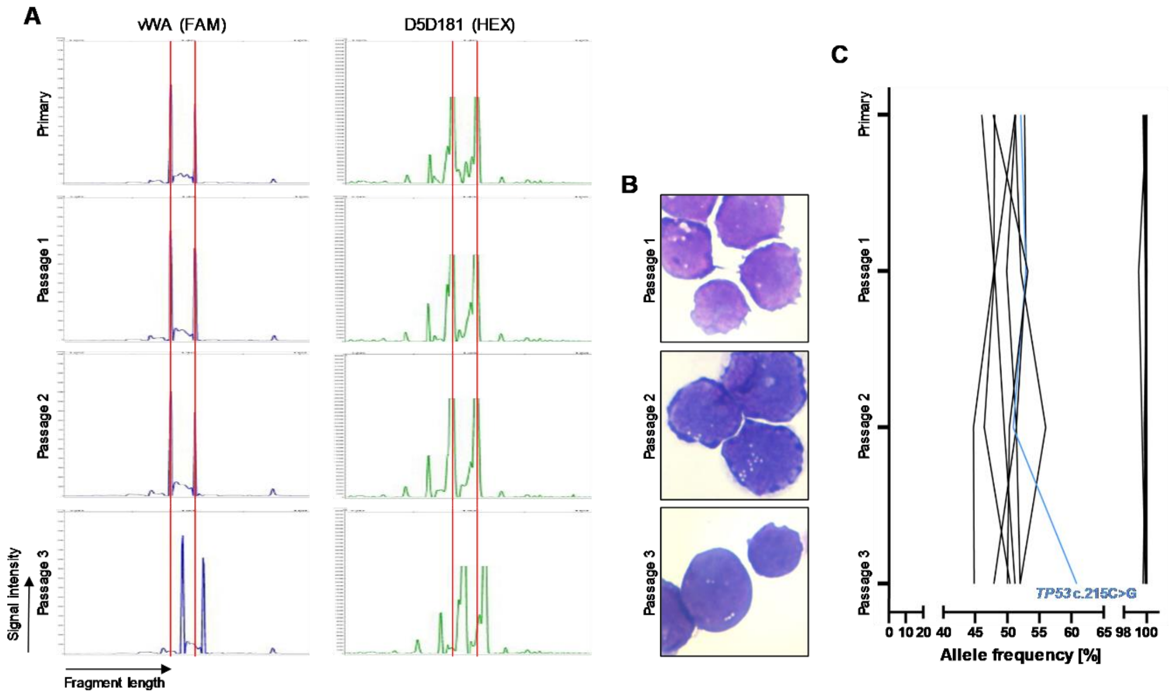

Figure S6. Molecular and morphological changes in patient #0134-derived tumor cells after serial transplantation. (**A**) Electropherograms of primary sample as well as passages 1, 2 and 3 for STR markers vWA and D5D181. The fragment length is distributed on the x axis while the y axis indicates signal intensity. The red lines indicate the annotated peaks in the primary sample. (**B**) The blast

morphology of bone marrow-derived cells was determined after cytospin preparation and Pappenheim staining. Representative cells were captured at 100-fold magnification using the EVOS xl core imaging system. (**C**) Genetic variants in primary sample and xenografts were detected using the Cancer Hotspot Panel v2 (Ion PGM System, Thermo Fisher Scientific). Fourteen variants were detected in the primary material and present in all xenografted samples. The graph illustrates the allelic frequency of each variant throughout serial transplantation with the only mutation with a relevant change, a *TP53* c.215C > G conversion, highlighted in blue.

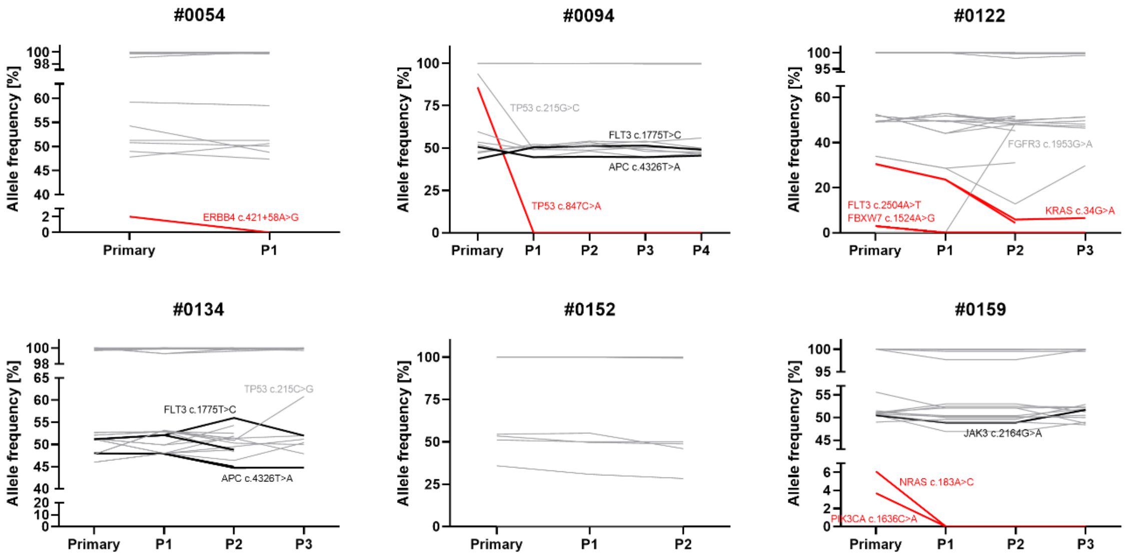

Figure S7. Presence of single nucleotide variants in *KMT2A* positive primary samples and consecutive PDX passages. All variants detected using the Ion AmpliSeq™ Cancer Hotspot Panel v2 (Thermo Fisher Scientific) are summarized irrespectively of their pathogenicity. Grey lines represent germline variants while red and black lines correspond to somatic or likely somatic variants with or without changes in allele frequency, respectively. Two individual mice were analyzed in passage 2 of patients #0122 and #0134 and depicted by separate lines. Only cells from one of those mice were used for subsequent xenografting to generate passage 3. P, passage.

Table S1. Total numbers of animals per passage for each primary sample and respectively injected cell counts.

**Laboratory ID**	**Cytogenetic Aberrations**	**Passage 1**		**Passage 2**		**Passage 3**		**Passage 4**		
		No of Mice	Cells Injected	No of Mice	Cells Injected	No of Mice	Cells Injected	No of Mice	Cells Injected	**Total**
0054	KMT2A	3	1.00E+06	3	2.50E + 06	2	2.50E + 06			8
0094	KMT2A	3	2.90E + 06	1	2.50E + 06	2	2.50E + 06	3	2.50E + 06	9
0122	KMT2A	2	3.70E + 06	2	2.50E + 06	2	4.00E + 05	2	2.50E + 06	8
0134	KMT2A	4	3.50E + 06	2	2.50E + 06	3	2.50E + 06			9
0152	KMT2A	1	1.90E + 06	4	2.50E + 06	4	1.85E + 06			9
0159	KMT2A	1	3.00E + 06	2	2.50E + 06	2	1.73E + 06	1	1.00E + 06	6
0043	BCR::ABL1	3	2.50E + 06	3	2.50E + 06	2	2.50E + 06			8
0141	BCR::ABL1	1	1.00E + 06	3	2.50E + 06	2	2.50E + 06			6
0151	BCR::ABL1	1	1.00E + 06	3	2.50E + 06	2	2.50E + 06			6
0200	BCR::ABL1	2	2.50E + 06	2	2.50E + 06	2	2.50E + 06			6
0212	BCR::ABL1	3	2.50E + 06	2	2.00E + 06	2	2.50E + 06			7
0019	T-ALL	1	1.00E + 06	2	2.50E + 06	2	2.50E + 06			5
0170	T-ALL	4	1.70E + 06	2	2.50E + 06	2	2.50E + 06			8
0202	none	2	2.50E + 06							2
P67	none	1	8.20E + 05	2	2.50E + 06					3
P33	none	3	1.40E + 06							3
0074	other	2	1.10E + 06							2
